# A comparative genomics screen identifies a *Sinorhizobium meliloti* 1021 *sodM*-like gene strongly expressed within host plant nodules

**DOI:** 10.1186/1471-2180-12-74

**Published:** 2012-05-15

**Authors:** Clothilde Queiroux, Brian K Washburn, Olivia M Davis, Jamie Stewart, Tess E Brewer, Michael R Lyons, Kathryn M Jones

**Affiliations:** 1 Department of Biological Science, Florida State University, Biology Unit I, 230A, 89 Chieftain Way, Tallahassee, FL, 32306-4370, USA; 2 Present address: Department of Psychology, Brooklyn College, Brooklyn, NY, USA; 3 Present address: Arthrex Orthopaedic Products, Jacksonville, FL, USA

**Keywords:** Rhizobia, *Sinorhizobium meliloti*, Alfalfa, Symbiosis, Nitrogen fixation, Bacteria, Legume, Genomics, α-proteobacteria

## Abstract

**Background:**

We have used the genomic data in the Integrated Microbial Genomes system of the Department of Energy’s Joint Genome Institute to make predictions about rhizobial open reading frames that play a role in nodulation of host plants. The genomic data was screened by searching for ORFs conserved in α-proteobacterial rhizobia, but not conserved in closely-related non-nitrogen-fixing α-proteobacteria.

**Results:**

Using this approach, we identified many genes known to be involved in nodulation or nitrogen fixation, as well as several new candidate genes. We knocked out selected new genes and assayed for the presence of nodulation phenotypes and/or nodule-specific expression. One of these genes, SMc00911, is strongly expressed by bacterial cells within host plant nodules, but is expressed minimally by free-living bacterial cells. A strain carrying an insertion mutation in SMc00911 is not defective in the symbiosis with host plants, but in contrast to expectations, this mutant strain is able to out-compete the *S. meliloti* 1021 wild type strain for nodule occupancy in co-inoculation experiments. The SMc00911 ORF is predicted to encode a “SodM-like” (superoxide dismutase-like) protein containing a rhodanese sulfurtransferase domain at the N-terminus and a chromate-resistance superfamily domain at the C-terminus. Several other ORFs (SMb20360, SMc01562, SMc01266, SMc03964, and the SMc01424-22 operon) identified in the screen are expressed at a moderate level by bacteria within nodules, but not by free-living bacteria.

**Conclusions:**

Based on the analysis of ORFs identified in this study, we conclude that this comparative genomics approach can identify rhizobial genes involved in the nitrogen-fixing symbiosis with host plants, although none of the newly identified genes were found to be essential for this process.

## Background

*Sinorhizobium meliloti* 1021 is a soil bacterium that establishes a nitrogen-fixing symbiosis with the host plants *Medicago sativa* (alfalfa) and *Medicago truncatula* (reviewed in [1,2]). These plants are not only agriculturally important, but are also key model organisms for studying the symbiotic interaction between rhizobial bacteria and their plant hosts. The goals of this study are to increase our understanding of this process and provide practical insights that may lead to the production of more efficient symbiotic strains of rhizobia. Increasing the efficiency of symbiotic nitrogen fixation is important in that it reduces the need for industrial production of nitrogen fertilizers, which is extremely costly in terms of petroleum and natural gas. In 2007, the US applied 13 million tons of industrially-produced nitrogen fertilizer to crops [3]. Fertilizers continue to be used to increase yields of legume crops [3], demonstrating that there is considerable room for improvement in these symbiotic associations.

*S. meliloti* fixes nitrogen in root nodules formed by the host plant, converting dinitrogen gas to ammonia. The development of these nodules requires that several signals be exchanged between the plant and the rhizobial bacteria. Flavonoid compounds produced by host plants signal *S. meliloti* to produce lipochitooligosaccharides called Nod factors (NFs) [4]. NF activates multiple responses in host plants, including tight curling of root hairs that traps bacterial cells within the curl, and cell divisions in the root cortex, which establish the nodule primordium [5,6]. The bacteria invade and colonize the roots through structures called infection threads, which originate from microcolonies of bacteria trapped in the curled root hair cells [1,7]. New infection threads initiate at each cell layer, eventually delivering the bacteria to the inner plant cortex [7]. There, the rhizobial bacteria are endocytosed by root cortical cells within individual compartments of host-cell membrane origin [2,8]. Within these compartments, signals provided by the plant and the low-oxygen environment induce the bacteria to differentiate into a form called a “bacteroid”, and to begin expressing nitrogenase, the nitrogen-fixing enzyme, and other factors that are required for the symbiosis [9,10].

Rhizobial fixation of dinitrogen requires not only the expression of nitrogenase (encoded by the genes *nifK* and *nifD*[11]), but also the assembly of cofactors and large inputs of energy and reductant [12]. Nitrogen fixation also requires a nitrogenase reductase, encoded by *nifH*[11]; iron-molybdenum cofactor biosynthesis proteins, encoded by *nifBnifE* and *nifE*; and electron transfer flavoproteins and ferredoxins (*fixA, fixB, fixC, fixX*) [13-16]. Bacteroids also increase their respiration rate, increasing the expression of the *fixNOQP* cytochrome c oxidase operons [17-20].

Many of the proteins required for nitrogen fixation are tightly regulated by oxygen-sensing systems and are produced by rhizobial bacteria only when they encounter a low-oxygen environment [21]. Nitrogenase and some of the other factors involved in nitrogen fixation are extremely oxygen-sensitive [22], thus their expression under inappropriate conditions would be ineffective. Even under microaerobic conditions, most rhizobial bacteria are not capable of nitrogen fixation in the free-living state [23]. The reasons for this are not completely understood, though it is known that legumes of the inverted repeat-lacking clade (IRLC), such as alfalfa and *M. truncatula,* which form indeterminate-type nodules, impose a specific differentiation program on the intracellular bacteria, most likely through the activity of plant-produced bioactive peptides [9,24]. Bacteroids also receive nutrients from the host plant, such as the carbon source malate [25-27]. Multiple bacterial cellular processes and differentiation programs contribute to the success of the symbiosis with host plants, and one of our goals is to use comparative genomics to predict previously uncharacterized *S. meliloti* open reading frames (ORFs) that may be involved in these processes, to test these predictions, and understand the mechanisms involved. In other bacterial species, comparative genomics of bacterial strains has been useful in finding new genes that are involved in metabolic pathways and in identifying virulence factors that distinguish pathogenic strains from commensal strains (examples include: [28,29]). In this study, a comparison of ORFS from nitrogen-fixing, plant-host nodulating rhizobia with closely-related non-nitrogen-fixing bacteria has identified ORFs that are expressed by *Sinorhizobium meliloti* within host plant nodules.

## Methods

### Genome comparisons

Searches were conducted at the Department of Energy Joint Genome Institute’s Integrated Microbial Genomes website, http://img.jgi.doe.gov/cgi-bin/pub/main.cgi. All of the genomes to be compared were selected from the genome display under the “Find Genomes” tab (see Table 1 for compared genomes). The selected genomes were saved. The “Phylogenetic profiler” for single genes was used to find genes in *Sinorhizobium/Ensifer meliloti* with homologs in the genomes to be intersected and without homologs in the genomes to be subtracted (see Table 1). The searches were conducted at 20–80% identity and the complete data output is listed in Additional file 1: Table S1.

**Table 1:** **Genome ORFs compared with*S. meliloti*1021**

**Genome**	**Subtracted or intersected**	**Lifestyle**
*Agrobacterium tumefaciens* C58 (Cereon) [30,31]	subtracted	plant pathogen
*Agrobacterium tumefaciens* C58 (Dupont) [30,31]	subtracted	plant pathogen
*Bartonella bacilliformis* KC583	subtracted	mammalian pathogen
*Bartonella henselae* Houston-1	subtracted	mammalian pathogen
*Bartonella quintana* Toulouse	subtracted	mammalian pathogen
*Bartonella tribocorum* CIP 105476	subtracted	mammalian pathogen
*Brucella abortus* bv 1 9-941	subtracted	mammalian pathogen
*Brucella canis* ATCC 23365	subtracted	mammalian pathogen
*Brucella melitensis* 16 M	subtracted	mammalian pathogen
*Brucella melitensis* bv Abortus 2308	subtracted	mammalian pathogen
*Brucella ovis* ATCC 25840	subtracted	mammalian pathogen
*Brucella suis* ATCC 23445	subtracted	mammalian pathogen
*Brucella suis* 1330	subtracted	mammalian pathogen
*Caulobacter crescentus* CB15 [32]	subtracted	free-living
*Caulobacter* sp. K31 [33,34]	subtracted	free-living
*Bradyrhizobium japonicum* USDA 110 [35]	intersected	nitrogen-fixing plant symbiont
*Mesorhizobium loti* MAFF303099 [36]	intersected	nitrogen-fixing plant symbiont
*Rhizobium etli* CFN 42 [37]	intersected	nitrogen-fixing plant symbiont
*Rhizobium leguminosarum* bv. viciae 3841 [38]	intersected	nitrogen-fixing plant symbiont
*Sinorhizobium medicae* WSM419 [39]	intersected	nitrogen-fixing plant symbiont

### Bacterial strains and growth conditions

*S. meliloti* 1021 strains were grown at 30°C in either LBMC (Luria Bertani [Miller] medium supplemented with 2.5 mM MgSO_4_ and 2.5 mM CaCl_2_), or 1/10 LB-7% sucrose medium, with 1 mM MgSO_4_ and 0.25 mM CaCl_2_, or M9 salts-10% sucrose medium, supplemented with 1 μg/mL biotin [40]. Bacterial plates contained 1.5% BactoAgar. Selections against strains carrying the *sacB* gene in the plasmid pK19mobsac were performed in M9 supplemented with 10% w/v sucrose or 1/10 LB-7% sucrose [41]. Appropriate antibiotics were used at the following concentrations for *S. meliloti* strains: streptomycin 500 or 1000 μg/mL; neomycin 200 μg/mL. *E. coli* strains were grown at 37°C in LB medium [40], with appropriate antibiotics used at the following concentrations: kanamycin 50 μg/mL; chloramphenicol 10 μg/mL.

### Construction of *S. meliloti* mutant strains

Mutant strains of *S. meliloti* 1021 with disruptions in ORFs described in Table 2 were constructed by amplifying internal ORF fragments using Phusion polymerase (New England Biolabs, Ipswich, MA, USA) and cloning into the plasmid pJH104, which carries a neomycin/kanamycin resistance marker (Jeanne Harris, Univ. Vermont, personal communication) [42]. Insertion of the pJH104 plasmid also creates transcriptional fusions to the *uidA* β-glucuronidase (GUS) gene. Non-disrupting GUS insertions of some ORFs (described in Table 2) were constructed by amplifying the entire ORF or operon and cloning the product into pJH104, and conjugating into *S. meliloti*. Deletion mutant strains were constructed by amplifying fragments flanking the ORF to be deleted and cloning the fragments into the *sacB* gene-containing suicide vector pK19mobsac [41]. (Some fragments were initially cloned into pCR-Blunt II-TOPO using the Zero-TOPO-Blunt cloning kit [Invitrogen, San Diego, CA, USA].) Mutant strains are listed in Table 2. Primers (Eurofins MWG Operon, Huntsville, AL, USA) and restriction enzymes (New England Biolabs, Ipswich, MA, USA) used for amplification and cloning of disruption, non-disrupting insertion, or deletion fragments are listed in Additional file 2: Table S2. Plasmids were mobilized into *S. meliloti* by triparental conjugation as described previously [43]. *S. meliloti* exconjugants were selected on LBMC medium containing 200 μg/mL neomycin and 1000 μg/mL streptomycin. Unmarked deletion strains were selected for loss of the *sacB* gene carried by the pK19mobsac vector by plating neomycin-resistant exconjugants to either M9 salts--10% sucrose medium or 1/10 LB-7% sucrose medium. Strains constructed by phage ϕM12 transduction of plasmid insertions into *S. meliloti* 1021 are denoted in the Tables as “Xsd”. Transductions using phage ϕM12 were performed according to published protocols [44]. For each mutant produced, at least two strains were isolated. For some of the mutants, including those which carry an unmarked ORF deletion, multiple independent isolates were obtained by selecting exconjugants from multiple independent conjugations. For most of the mutants carrying an insertion of the pJH104 plasmid, the independent isolates were the original isolate and strains constructed by transduction of the neomycin-resistance marker into wild type *S. meliloti* 1021 via phage ϕM12 [44]. 

**Table 2:** ***S. meliloti*1021-derived mutant strains**

**ORF**	**Predicted function**	**Length (amino acids)**	**Type of mutation**	**Strain name**
SMc01562	hypothetical protein	96	deletion	ΔSMc01562.6
				ΔSMc01562.25
				ΔSMc01562.100
SMc01562	hypothetical protein	96	non-disrupting insertion of pJH104 GUS marker	A104U.original
				A104U.Xsd1
				A104U.Xsd6
				A104U.Xsd25
				A104U.Xs100
SMc01986	hypothetical protein	119	deletion	ΔSMc01986.1
				ΔSMc01986.6
				ΔSMc01986.25
				ΔSMc01986.100
SMc01986	hypothetical protein	119	non-disrupting insertion of pJH104 GUS marker	C104.1A.Xsd1
				C104.1A.original
				C104.2B.Xsd100
SMc00135	hypothetical protein	243	deletion	ΔSMc00135.B1
				ΔSMc00135.B17
SMc00135	hypothetical protein	243	non-disrupting insertion of pJH104 GUS marker	B104.3A
				B104.4B
				B104.2 C
SMc01422	hypothetical protein (probable operon with SMc01423,SMc01424)	128	deletion (SMc01422, SMc01423, SMc01424 all deleted in this strain)	ΔSMc01422-24.D21 ΔSMc01422-24.D29
SMc01423	probable nitrile hydratase subunit β	219	deletion	same as above
SMc01424	probable nitrile hydratase subunit α	213	deletion	same as above
SMc01424-01422	hypothetical protein (probable operon with SMc01423,SMc01422)	213	non-disrupting insertion of pJH104 GUS marker	D104.2A
				D104.3B
				D104.1 C
SMa0044	hypothetical protein	89	deletion	ΔSMa0044.c1
				ΔSMa0044.c6
				ΔSMa0044.c10
SMa0044	non-disrupting insertion of pJH104 GUS marker	89		SMa0044.104.1A
				SMa0044.104.1B
				SMa0044.104.4 C
SMb20431	hypoth. arylmalonate decarboxylase	261	ORF-disrupting insertion of pJH104 GUS marker	SMb20431.original
				SMb20431.Xsd1
SMb20360	hypothetical protein	243	ORF-disrupting insertion of pJH104 GUS marker	SMb20360.original
				SMb20360.Xsd1
SMc03964	hypothetical protein	300	ORF-disrupting insertion of pJH104 GUS marker	SMc03964.original
				SMc03964.Xsd6
SMc00911	hypothetical protein	275	ORF-disrupting insertion of pJH104 GUS marker	SMc00911.original
				SMc00911.Xsd1
				SMc00911.original2
SMa1334	hypothetical protein	398	ORF-disrupting insertion of pJH104 GUS marker (may have a polar effect on 3′ genes Sma1332,-1331,-1329)	SMa1334.original
				SMa1334.Xsd1
SMc01266	hypothetical protein	438	ORF-disrupting insertion of pJH104 GUS marker (may have a polar effect on 3′ gene Smc01265)	SMc01266.original
				SMc01266.Xsd1
*greA*	transcription elongation factor	158	ORF-disrupting insertion of pJH104 GUS marker	greA.12.4.1a
*expA1 (wgaA)*	EPSII biosynthesis enzyme	490	ORF-disrupting insertion of Tn5-Nm in *expA*—symbiotically proficient, competitor assay strain	expA125::Tn5.Xsd1

### Plant nodulation assays

The host plant *Medicago sativa* (alfalfa) cv. Iroquois was prepared for inoculation with *S. meliloti* as in Leigh *et al.* (1985) with modifications: seeds were sterilized for 5 minutes in 50% bleach, rinsed in sterile water, and germinated for 3 days on 1% w/v plant cell culture-tested agar/water (Sigma, St. Louis, MO, USA) [45]. Seedlings were then moved to individual 100 mm x 15 mm Jensen’s medium plates [46], and inoculated with 100 μL of OD_600_ = 0.05 *S. meliloti* of the appropriate strain. Plants were grown in a Percival AR-36 L incubator (Perry, IA, USA) at 21°C, with 60–70% relative humidity, and 100–175 μmol m^−2^ s^−1^ light. Plants were measured at 5 weeks and 6.5 weeks of growth. t-tests (unpaired, two-tailed) were performed in Microsoft Excel and in GraphPad (http://www.graphpad.com/quickcalcs/ttest1.cfm?Format=C).

Nodulation competition assays were performed in the same way as the plant assays described above, except that strains to be tested in competition against one another were prepared as a mixed 1:1 inoculum immediately before inoculation. Bacteria were harvested from nodules after 5 or 6.5 weeks of growth by excising the nodules from roots, surface sterilizing in 20% bleach for 5 min., washing in sterile, distilled water, and crushing the nodules in 1.5 mL tubes with a micro-pestle (Kimble-Chase, Vineland, NJ), in LB + 0.3 M glucose [45]. Dilutions of the material from crushed nodules were plated on LBMC + 500 μg/mL streptomycin. Colonies were patched from these plates to LBMC + 500 μg/mL streptomycin and 200 μg/mL neomycin to determine the fraction of bacteria that carry the neomycin-resistance marker in the insertion plasmid pJH104.

### Detection of β-glucuronidase activity and imaging of root nodules

β-glucuronidase expression by bacteria within nodules was detected by excising nodules, surface sterilizing with 20% bleach for 5 min., rinsing in sterile water, and staining in X-gluc buffer (1 mM 5-bromo-4-chloro-3-indolyl-beta-D-glucuronic acid, cyclohexylammonium salt; 0.02% SDS; 50 mM Na-phosphate, pH 7) [47] for the amount of time indicated in Table 3. Whole nodules were imaged on an AZ100 Multi-Zoom Microscope equipped with a DS-Fi1, 5 Megapixel color camera (Nikon Instruments U.S., Melville, NY). β-glucuronidase expression by bacteria on LBMC plates was detected by streaking bacteria to plates that had been spread with 40 μL of X-gluc solution (100 mM 5-bromo-4-chloro-3-indolyl-beta-D-glucuronic acid, cyclohexylammonium salt solution in dimethylformamide). 

**Table 3:** Expression of β-glucuronidase (GUS) fusions

**ORF**	**strain**	**% of nodules with GUS expression**	**Strength of nodule GUS expression**	**Staining time**	**Pattern of nodule GUS expression**	**Free-living GUS expression**
**N/A**	*S. meliloti* 1021 wild type (negative control)	0/39 = 0%	−	variable	none	−
**SMc00911**	SMc00911.original	18/20 = 90%	++++	1.5–3.75 hr	whole nodule	+
	SMc00911.Xsd1	18/18 = 100%	++++	1.5–3.75 hr	whole nodule	n.d.
	SMc00911.original2	n.d.	n.d.	N/A	N/A	+
**SMb20360**	SMb20360.original	8/13 = 62%	++	3–5 hr	invasion zone-fixation zone	−
	SMb20360.Xsd1	13/16 = 81%	++	3–5 hr	invasion zone-fixation zone	−
**SMc00135**	B104.3A	6/8 = 75%	+	2–3 hr	invasion zone-interzone	+
	B104.4B	8/8 = 100%	+	2–3 hr	invasion zone-interzone	++
	B104.2 C	6/8 = 75%	++	2–3 hr	invasion zone-interzone	++
**SMc01562**	A104U.original	7/8 = 88%	+	4–6 hr	interzone	−
	A104U.Xsd1	3/7 = 43%	+/−	4–6 hr	interzone-fixation zone	n.d.
	A104U.Xsd6	8/8 = 100%	+	4–6 hr	interzone-fixation zone	n.d.
	A104U.Xsd25	3/8 = 38%	+/−	4–6 hr	interzone-fixation zone	n.d.
	A104U.Xs100	4/9 = 44%	+	4–6 hr	fixation zone	n.d.
**SMc01266**	SMc01266.original	13/18 = 72%	+	3 hr	invasion zone-fixation zone	+/−
	SMc01266.Xsd1	13/18 = 72%	++	3 hr	invasion zone	−
**SMc03964**	SMc03964.original	8/15 = 53%	++	3–5 hr	interzone	+/−
	SMc03964.Xsd6	9/19 = 47%	++	3–5 hr	interzone-fixation zone	−
**SMc01424-22**	D104.2A	0/8 = 0%	−	4–6 hr	N/A	+/−
	D104.3B	7/8 = 88%	++	4–6 hr	invasion zone-interzone	+/−
	D104.1 C	6/8 = 75%	+	4–6 hr	invasion zone-fixation zone	+/−
**SMa0044**	SMa0044.104.1A	4/8 = 50%	+/−	6–7 hr	invasion zone-interzone	+++
	SMa0044.104.1B	4/8 = 50%	+/−	6–7 hr	interzone	+++
	SMa0044.104.4 C	4/8% 50%	+/−	6–7 hr	interzone	+++
**SMb20431**	SMb20431.original	10/16 = 63%	+	5–12 hr	invasion zone-fixation zone	−
	SMb20431.Xsd1	11/15 = 73%	+	5–12 hr	interzone	−
**SMc01986**	C104.1A.Xsd1	0/6 = 0%	−	24 hr	N/A	n.d.
	C104.1A.original	n.d.	n.d.	24 hr	n.d.	+/−
	C104.2B.Xsd100	2/18 = 11%	+/−	24 hr	fixation zone	n.d.
**SMa1334**	SMa1334.original	0/11 = 0%	−	5–24 hr	N/A	−
	SMa1334.Xsd1	0/13 = 0%	−	5–24 hr	N/A	−

## Results

### Comparisons of *Sinorhizobium meliloti* open reading frames with those of other rhizobia and with non-nitrogen fixing α-proteobacteria

Rhizobial functions required for symbiotic nitrogen fixation with legume plants have typically been discovered through the classical bacterial genetic technique of transposon mutagenesis, followed by screening mutants for loss of symbiotic function. We have used an alternative comparative genomics strategy to search for rhizobial genes involved in symbiosis. In this approach, searches of the Joint Genome Institute, Integrated Microbial Genomes (JGI IMG) system [48] were performed to find ORFs that *S. meliloti* 1021 shares with the symbiotic nitrogen-fixing α-proteobacteria (α-rhizobia) *S. medicae* WSM419, *Rhizobium etli* CFN 42, *Rhizobium leguminosarum* bv. viciae, *Mesorhizobium loti* MAFF303099, and *Bradyrhizobium japonicum* USDA110. A novel aspect of this strategy is that these searches were restricted by prior elimination of all *S. meliloti* ORFs that are present in any of 15 non-nitrogen-fixing, non-symbiotic α-proteobacteria (species listed in Table 1). (See Materials and Methods for search procedure.) The genomes used in the analysis were chosen based on the rhizobial genomes available in the JGI IMG database when the analysis was initially performed. The searches were conducted at multiple identity levels (20%–80%), and the output data from all the searches is presented in Additional file 1: Table S1. The genome subtractions eliminated genes common to α-proteobacteria with non-symbiotic lifestyles. For example, a search conducted at 50% identity, intersecting the *S. meliloti* ORFs with homologs in the 5 α-rhizobia species yields 1281 genes. However, when the search for homologs is conducted with subtraction of the ORFs from the 15 non-rhizobial species, the search yield is 58 genes ( Additional file 3: Table S3).

The result of the searches was a list of 139 ORFs common to the α-rhizobia (listed in Additional file 3: Table S3), but not found in the non-nitrogen-fixing, non-symbiotic α-proteobacteria. Among these 139 ORFs were 11 genes known to be involved in nitrogen fixation (Table 4 and Additional file 3: Table S3), including: *nifHnifDnifKnifBnifEnifNfixAfixB*, and *fixC* (see Introduction) and 8 known to be involved in Nod factor production, including *nodAnodBnodCnodJ* and *nodI*[5], thus 13.7% (19/139) of the ORFs selected by this comparative gemonics approach are already known to be important for symbiotic function. 

**Table 4:** **Function distribution of the 139 ORFs from genome searches (SeeAdditional file**3: Table S3**for complete gene list)**

**Function**	**Number of ORFs**
Nitrogen fixation	11
Nod factor production/modification	8
Transposase	10
Predicted transcriptional regulator	8
Predicted transport protein	14
Predicted adenylate/guanylate cyclase	7
Other predicted function	37
Hypothetical protein	44

There were also 44 hypothetical proteins/proteins of unknown function among the 139 ORFs detected in the comparative genomic screen. The predicted functions of the remaining ORFs included transposases*,* transcriptional regulators, transport proteins, and adenylate/guanylate cyclases (Table 4). These are classes of genes that may participate in many of the functions that distinguish α-rhizobia from their non-symbiotic α-proteobacterial relatives, such as signaling to the host plant, reprogramming their metabolism for nitrogen fixation, and importing specific nutrients and differentiation signals from the plant [9,10,49]. Also, atypical adenylate cyclases have been noted before in the rhizobia [50].

### Construction and symbiosis assays of mutants in conserved genes

Thirteen of the 139 conserved ORFs were chosen for further study because they are of undetermined function in *S. meliloti* and have no close homologs in the *S. meliloti* genome that might be expected to provide redundant function. Six of the longer ORFs, including SMc00911, were disrupted by cloning a small internal ORF fragment into the plasmid pJH104, conjugating the plasmid into *S. meliloti* 1021, and selecting for single-crossover insertion/disruption mutants. ( Additional file 2: Table S2 lists primer sequences and disruption fragment sizes and positions.) For the 6 remaining ORFs, 3 that are under 750 bp long (SMc01562, SMc01986 and SMc00135) and 3 that are all in a single operon (SMc01424, SMc01423, and SMc01422), deletion was judged to be a better strategy than disruption. SMc01424, SMc01423, and SMc01422 were all deleted as a single segment from the start codon of SMc01424 to the stop codon of SMc01422. The endpoints of the individual deletions of SMc01562, SMc01986, and SMc00135 were dictated by the position of the most suitable PCR primers. ( Additional file 2: Table S2 lists primer sequences and deletion sizes and positions.) Either the disruption or the deletion strategy is expected to result in a strain that does not produce a full-length version of the protein encoded by that ORF. These ORFs and the insertion and/or deletion mutant strains of each are listed and described in Table 2. The resulting mutant strains were then tested for symbiotic proficiency on the host plant alfalfa.

**Figure 1:**
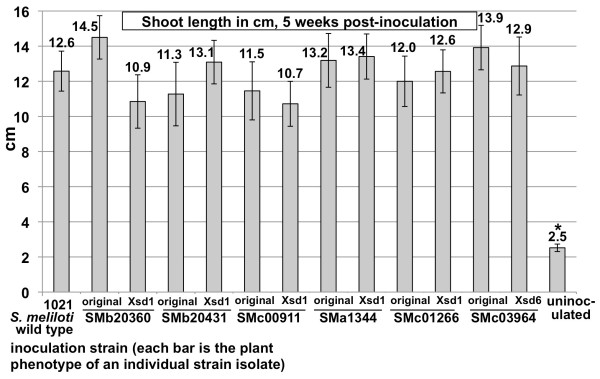
**Plant shoot length in cm, 5 weeks after inoculation with insertion mutant strains (mutant strain information is summarized in Table **3**).** For each of the 6 ORF disruptions, the plant phenotype of the original isolate and that of a phage ϕM12 transductant of that strain are shown. Mean values are given above graph bars. Error bars represent standard error of the mean. Asterisks indicate samples with mean heights significantly different from the wild type. The number of plants tested and the number of nodules/plant for these assays are presented in Table 4.

**Figure 2:**
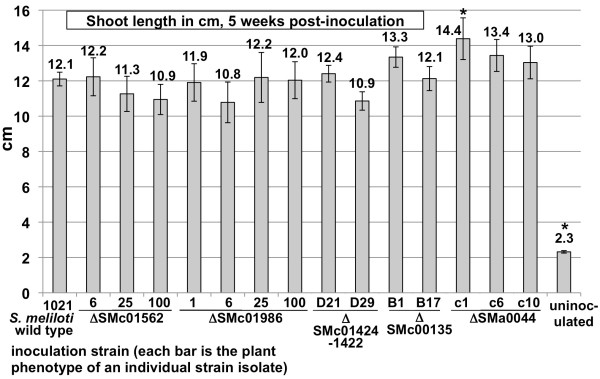
**Plant shoot length in cm, 5 weeks after inoculation with deletion mutant strains (summarized in Table **3**).** For each of the ORF deletions, the plant phenotype of at least two isolates/and or transductants of each strain are shown. Mean values are given above graph bars. Error bars represent standard error of the mean. Asterisks indicate samples with mean heights significantly different from the wild type. The number of plants tested and the number of nodules/plant for these assays are presented in Table 4.

For the initial phenotypic analysis, the ability of the mutants to successfully provide the plants with fixed nitrogen was determined. Alfalfa plants were inoculated with the bacterial mutants and after 5 weeks of growth, the shoot length attained on nitrogen-free medium was compared with plants inoculated with the *S. meliloti* 1021 wild type as the positive control and uninoculated plants as the negative control. Figure 1 shows the shoot length of alfalfa plants inoculated with wild type *S. meliloti* 1021 or with disruption mutant strains of the ORFs SMb20360, SMb20431, SMc00911, SMa1344, SMc01266, and SMc03964. Alfalfa plants inoculated with these strains attain a similar average shoot length as that of the wild type, demonstrating that all of these strains are able to form a successful symbiosis with this host plant. Figure 2 presents the same type of assay as Figure 1 for deletion mutants in the ORFs SMc01562, SMc01986, SMc01424-22, SMc00135, and SMa0044. Additional data on the plant assays in Figures 1 and 2 is presented in Table 5. The number of plants inoculated with each strain, the average number of mature, pink nodules per plant and the average number of white pseudonodules per plant are shown. All of these mutant strains are able to mount a successful symbiosis with the host plant alfalfa.

**Table 5:** Mean nodule number

**ORF**	**Strain name**	**Number of alfalfa plants tested**	**Mean number pink nodules/ plant ± std. error**	**Mean number white pseudonodules/plant ± std. error**
**N/A**	*S. meliloti* 1021 wild type, data set 1 (see Figure 1)	9	11.9 ± 1.0	3.2 + 1.2
**SMb20360**	SMb20360.original	8	17.4 ± 2.5	4.5 ± 1.2
	SMb20360.Xsd1	10	14.7 ± 1.7	4.4 ± 1.4
**SMb20431**	SMb20431.original	11	12.8 ± 1.6	3.0 ± 0.6
	SMb20431.Xsd1	11	13.3 ± 1.9	3.8 ± 0.8
**SMc00911**	SMc00911.original	11	14.3 ± 2.5	3.3 ± 0.8
	SMc00911.Xsd1	11	15.3 ± 1.8	3.2 ± 1.1
**SMa1334**	SMa1334.original	10	15.7 ± 2.1	5.7 ± 0.9
	SMa1334.Xsd1	11	16.4 ± 1.1	3.6 ± 1.7
**SMc01266**	SMc01266.original	11	14.4 ± 2.4	4.2 ± 0.5
	SMc01266.Xsd1	11	17.8 ± 1.6	4.6 ± 1.2
**SMc03964**	SMc03964.original	11	16.3 ± 1.6	4.2 ± 0.5
	SMc03964.Xsd6	10	15.2 ± 2.3	4.0 ± 0.9
**N/A**	uninoculated, data set 1 (see Figure 1)	5	0	0
**N/A**	*S. meliloti* 1021 wild type, data set 2 (see Figure 2)	179	12.5 ± 0.5	3.2 ± 0.3
**SMc01562**	ΔSMc01562.6	24	14.1 ± 1.3	2.2 ± 0.4
	ΔSMc01562.25	25	11.6 ± 1.2	2.5 ± 0.5
	ΔSMc01562.100	24	11.8 ± 0.9	2.0 ± 0.6
**SMc01986**	ΔSMc01986.1	26	18.0 ± 1.8	4.5 ± 0.8
	ΔSMc01986.6	26	15.3 ± 2.1	4.4 ± 0.8
	ΔSMc01986.25	25	17.2 ± 2.3	6.8 ± 1.1
	ΔSMc01986.100	25	16.8 ± 1.8	6.7 ± 1.0
**SMc01424-22**	ΔSMc01422-24.D21	110	13.1 ± 0.7	3.7 ± 0.4
	ΔSMc01422-24.D29	109	11.1 ± 0.6	3.6 ± 0.3
**SMc00135**	ΔSMc00135.B1	81	14.0 ± 0.7	2.8 ± 0.3
	ΔSMc00135.B17	76	13.5 ± 0.9	3.3 ± 0.4
**SMa0044**	ΔSMa0044.c1	24	11.8 ± 1.3	4.2 ± 0.6
	ΔSMa0044.c6	25	12.6 ± 1.2	3.0 ± 0.8
	ΔSMa0044.c10	24	13.5 ± 1.2	2.0 ± 0.5
**N/A**	uninoculated, data set 2 (see Figure 2)	82	0	0.1 ± 0.1

### SMc00911 is the most strongly expressed in the nodule of the conserved ORFS

**Figure 3:**
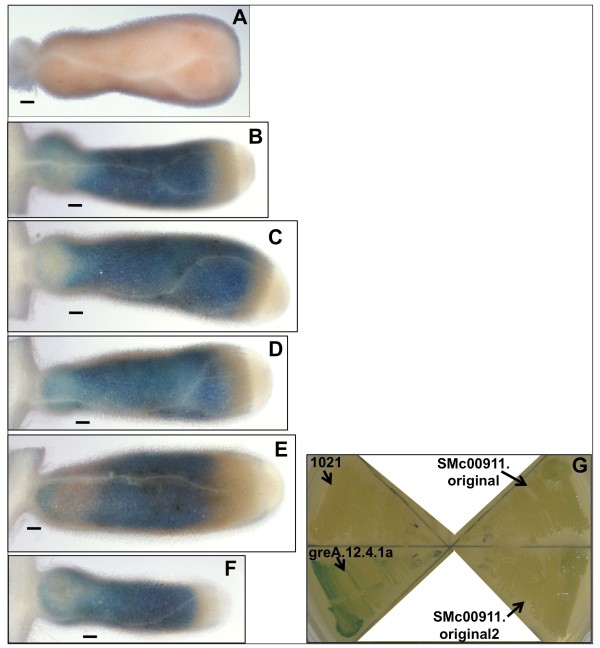
**Expression of** β**-glucuronidase (GUS)-encoding reporter gene* uidA *inserted within SMc00911.***S. meliloti* within alfalfa root nodules (**B**–**F**) express GUS inserted in SMc00911 throughout the nodule. Panel **A** shows an alfalfa nodule invaded by wild type *S. meliloti* 1021 that does not express GUS (subjected to the same staining procedure as **B**–**F**). (Roots in B, C, and D were inoculated with strain SMc00911. Xsd1. Roots in E and F were inoculated with strain SMc00911.original.) Nodules were stained for 3.75 hr after 5 weeks of growth post-inoculation. Scale bars correspond to 0.1 mm. Panel **G** shows SMc00911-controlled GUS expression in *S. meliloti* grown on solid LBMC medium. Wild type *S. meliloti* 1021 is shown as a negative control for GUS expression and a strain carrying the same GUS insertion plasmid in the *greA* gene is shown as a positive control for GUS expression in free-living cells. Strain SMc00911.original and a ϕM12 transductant of this strain were tested on plants.

**Figure 4:**
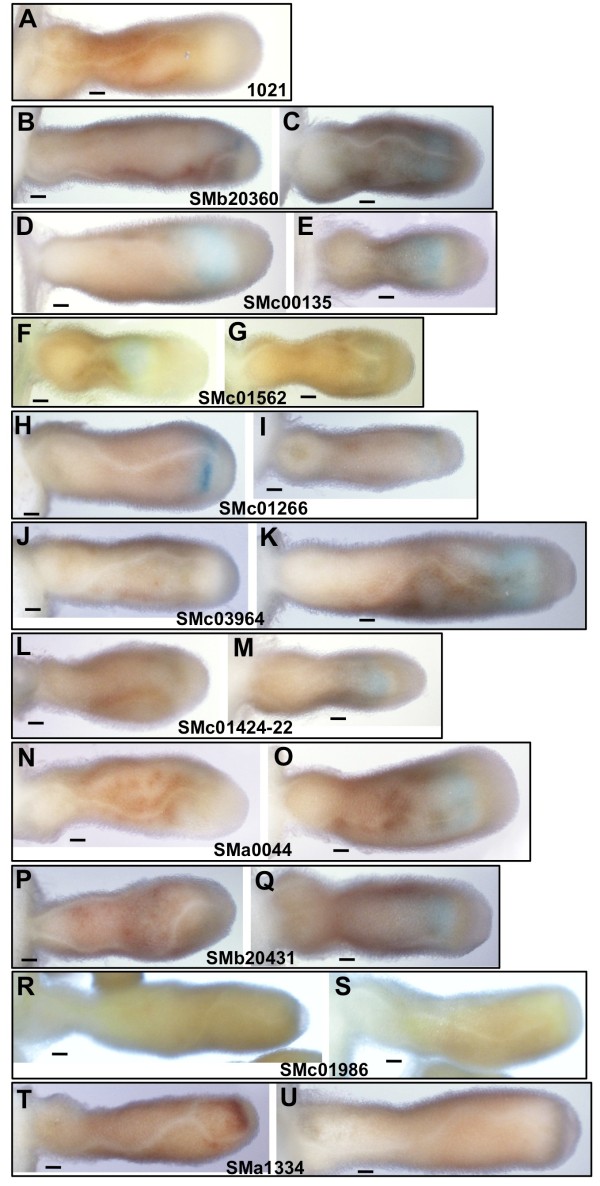
**Expression of β-glucuronidase (GUS)-encoding gene* uidA *expressed under the control of the promoter elements of the following ORFs: SMb20360 (B and C); SMc00135 (D and E); SMc01562 (F and G); SMc01266 (H and I); SMc03964 (J and K); SMc01424-22 (L and M); SMa0044 (N and O); SMb20431 (P and Q); SMc01986 (R and S); SMa1334 (T and U).** SMb20360 and SMc00135 are strongly expressed in the nodules. (See Table 3 for percentage of nodules with GUS expression and staining times.) SMc01562, SMc01266, SMc03964 and the SMc01424-22 operon are expressed at a moderate level in the nodules. The remaining ORFs are expressed at a very low level in the nodule (or not at all). *S. meliloti* 1021 wild type is shown in Panel **A** as a negative control for GUS expression. Scale bars correspond to 0.1 mm.

To determine if the 13 ORFs analyzed in this study might play a role in symbiosis, despite the fact that they are not strictly required for symbiosis, the expression pattern of each of these ORFs was determined both for bacteria within the nodule and in the free-living state. The SMc00911 ORF is very strongly expressed by bacteria within the nodule (Figure 3B–F), but it expressed at a very low level by free-living bacteria on LBMC plates (Figure 3G). The nodules shown in Figure 3 are expressing β-glucuronidase (GUS) from a pJH104 plasmid insertion in Smc00911. The nodules shown were stained for 3.75 hr. There is strong staining throughout the nodule, with slightly weaker staining at the invasion zone near the distal end of the nodule. The nodule expression of the SMc00911::GUS fusion is much stronger than the expression of any of the other fusions tested (see Figure 4 and Table 3). In contrast, SMc00911 is expressed at a very low level by free-living *S. meliloti* carrying the SMc00911::GUS fusion grown on LBMC plates (Figure 3G and Table 3). For comparison, Figure 3G also shows that a *greA*::GUS fusion strain of *S. meliloti* constructed with the same reporter insertion plasmid, pJH104, is strongly expressed under these conditions. Table 3 summarizes the expression data for all of the GUS fusion strains.

Two of the other ORFs tested, SMb20360 and Smc00135, are also strongly expressed in nodules (Figure 4B–E, Table 3), and another six, SMc01562, SMc01266, SMc03964 and the three ORFs in the SMc01424-22 operon are moderately expressed (Figure 4F–M, Table 3). Of these, only SMc00135 is expressed at approximately the same level by bacteria within the nodule and by free-living bacteria ( Additional file 4 and Additional file 5 show images of the free-living expression of GUS fusions of all the ORFs tested). However, none of the other ORFs that are expressed in the nodule are expressed as strongly as SMc00911 (Figure 3 and Figure 4). Two of the ORFs, SMa0044 and SMb20431, are expressed at a very low level in the nodule, and no nodule expression was detected for SMc01986 and SMa1334 (Figure 4). Sma0044 has an unusual expression pattern in that it is expressed strongly by free-living bacteria (Additional file 5A), but its expression appears to be much reduced in the nodule (Figure 4N–O).

Because of the strong expression of SMc00911 by bacteria in the nodule, the SMc00911 mutant strains were chosen for further study in competition experiments (see below).

### An insertion mutant of SMc00911 out-competes the *S. meliloti* 1021 wild type for nodule occupancy

Many *S. meliloti* mutant strains that are able to form a successful symbiosis when singly inoculated on host plants are deficient in the ability to successfully compete for nodule occupancy against the wild type strain in a mixed infection [42,51]. Competitive nodulation experiments are likely to be a better approximation of the situation that rhizobial bacteria encounter in the soil, where they may be competing against several different rhizobial strains for host plant invasion and nodule occupancy. The SMc00911 insertion mutant strains were chosen for competition analysis because this ORF is strongly expressed in the nodule and these strains might be expected to be at a competitive disadvantage in the absence of the full-length SMc00911 protein. However, in contrast to expectations, the SMc00911 insertion mutant strains strongly out-compete the *S. meliloti* 1021 wild type strain for nodule occupancy in a mixed 1:1 infection (Table 6). Of the nodules tested from plants inoculated with a 1:1 mixture of 1021 wild type and an SMc00911 insertion mutant, all of the nodules were colonized by either the SMc00911 insertion mutant alone or by a mixture of the mutant and the wild type (Table 6). Less than 22% of the mixed-inoculum nodules were colonized by 1021 wild type alone. Also, all of the mixed nodules contained a larger proportion of SMc00911 insertion mutant bacteria than 1021 wild type bacteria (Table 6). The recovered bacteria from one of the 8 nodules that had been inoculated with the SMc00911.Xsd1 strain alone included a small number of neomycin-sensitive colonies (Table 6, line 3). This suggests that the gene disruption plasmid inserted in the SMc00911 ORF is lost by bacteria in the nodule at a very low rate. Taken together, these competition results suggest that disruption of the SMc00911 ORF actually confers a competitive advantage to *S. meliloti* in the symbiosis with host plants. The SMc00911 ORF is predicted to encode a 275 amino acid protein with a rhodanese-like sulfurtransferase domain from amino acids 7–100 and a chromate-resistance protein domain from amino acids 122–256 [52]. The SMc00911 mutants carry the pJH104-GUS-expression/disruption plasmid inserted at nucleotide position 597 out of 828 total nucleotides, which would result in the production of a truncated protein containing only amino acids 1–199, based on the *S. meliloti* 1021 genome sequence [53,54]. Thus the SMc00911 insertion mutants are predicted to produce a protein that contains the whole rhodanese-like sulfurtransferase domain, but only a portion of the chromate-resistance protein domain. 

**Table 6:** **SMc00911-disruption strains out-compete*S. meliloti*1021 wild type for nodule occupancy**

**Inoculum**	Number of nodules tested*	**Number of nodules containing no neomycin-resistant bacteria**	**Number of nodules containing only neomycin-resistant bacteria**	**Number of nodules containing a mixture of neomycin-resistant and sensitive bacteria**	**Average percent of neomycin-resistant bacteria in mixed nodules**
*S. meliloti* 1021 wild type (neomycin-sensitive)	8	4 = 100%	0 = 0%	0 = 0%	N/A
SMc00911.original (neomycin-resistant)	16	0 = 0%	16 = 100%	0 = 0%	N/A
SMc00911.Xsd1 (neomycin-resistant)	16	0 = 0%	15 = 93.8%	1 = 6.3%	95.2% ± 0.00%
SMc00911.original:1021—mixed 1:1	32	7 = 21.9%	18 = 56.3%	7 = 21.9%	67.4% ± 14.2%
SMc00911.Xsd1:1021—mixed 1:1	31	2 = 6.5%	21 = 67.7%	8 = 25.8%	76.7% ± 9.8%

* 1–2 nodules/plant were analyzed.

In contrast to the SMc00911 insertion mutants, deletion mutants of SMc01562 (which is expressed in the nodule, but at a much lower level than SMc00911 (Figure 4)) are able to compete as effectively as *S. meliloti* 1021 wild type against a competitor assay strain carrying a neomycin-resistance marker (data not shown), suggesting that the loss of this protein confers neither a symbiotic disadvantage nor an advantage to *S. meliloti* 1021.

## Discussion

### Smc00911, a conserved rhizobial ORF expressed strongly in the nodule

Our comparative genomics screen has identified an *S. meliloti* 1021 ORF (SMc00911) that is strongly expressed within host plant nodules, but is expressed in the free-living state at a very low level. Surprisingly, disruption of this ORF confers a competitive advantage for nodule occupancy on *S. meliloti* 1021. Smc00911 is predicted to encode a 275 amino acid protein with overall similarity to SodM-like (superoxide dismutase-like) proteins [55,56]. There are 57 “SodM-like proteins” with >40% identity to SMc00911 in the NCBI database [56]. SMc00911 contains two distinct, conserved domains: a 94 amino acid domain (amino acids 7–100) similar to the GlpE sufurtransferase/rhodanese homology domain (cd01444), and a 135 amino acid (amino acids 122–256) chromate-resistance-exported protein domain (pfam09828) [52]. The SMc0911 mutant strains constructed in this study are predicted to produce a protein consisting of the first 199 amino acids of the full-length protein plus four amino acids encoded by the multiple cloning site of pJH104, before encountering a stop codon (Melanie Barnett, Stanford University personal communication) [53,54]. This truncated protein product would include the entire rhodanese-homology domain and approximately half of the chromate-resistance protein domain. One possibility is that the competitive advantage that the SMc00911-insertion mutant strains have against the 1021 wild type strain is due to the expression of this truncated protein, rather than simply a loss-of-function of the full-length protein. Even though SMc00911 is annotated as a “SodM-like” protein in the NCBI database [53,54,56], there are only two short segments of similarity (8 amino acids [38% identity] and 11 amino acids [36% identity]) with a protein confirmed to be a SodM from *Xanthomonas campestris* pv. *campestris* (accession no. p53654) [57]. Thus, since the N-terminal similarity of SMc00911 to the GlpE sufurtransferase/rhodanese homology domain and the C-terminal similarity to the chromate-resistance protein domain are both greater than the similarity of this protein to SodM, “SodM-like” may not be the most-appropriate annotation for this ORF. There are two *sod* ORFs in the *S. meliloti* 1021 genome, *sodB* (SMc00043) (SMc02597) and a bacteriocuprein-family *sodC* (SMc02597) [2,53,54]. An *S. meliloti* 1021 *sodB* loss-of-function mutant forms a functional symbiosis with host plants [58], while the symbiotic phenotype of a *sodC* mutant has not been reported.

### Expression of other αhizobial conserved ORFS

Although they are not required for development of a functional symbiosis by *S. meliloti* 1021, the ORFs SMb20360 and SMc00135 are also strongly expressed in nodules, while SMc01562, SMc01266, SMc03964 and the SMc01424-22 operon are moderately expressed (Figure 4; Table 3). However, the expression of SMc00135 is not specific to the nodule (Figure 4 and Additional file 5). SMb20360 is predicted to encode a protein of the Clp-protease superfamily (COG0740), with specific similarity to ClpP [52]. Polar localization of the ClpXP protease complex within *S. meliloti* cells has been found to be important for *S. meliloti* bacteroid differentiation [59], and it is possible that ClpP proteases play a role in the bacteroid differentiation process. Interestingly, in another study, a signature-tagged mutant in SMb20360 was found to be highly competitive for survival, in the free-living state, in competition experiments under salt- and detergent-stressed conditions [60]. SMc01562 is predicted to encode a member of the GYD-domain containing protein superfamily (COG4274) [52]. No function has been reported for this protein family [56]. SMc01266 is predicted to encode a member of the Von Willebrand factor type A (vFWA) superfamily (cl00057), however proteins containing a vFWA domain participate in a wide variety of functions [61]. Expression of SMc01266 has previously been shown to increase in bacteroids [62] (reference Supplemental Dataset 3), and during phosphate stress [63]. Smc03964 is predicted to possess a twin-arginine export signal [64], and to encode a member of the metallophosphatase superfamily (cl13995), a group of phosphatases with diverse functions [52]. ORFs SMc01424, SMc01423, and SMc01422 appear to be part of a single operon and they encode, respectively, a predicted nitrile hydratase alpha subunit protein, a nitrile hydratase beta subunit protein, and a nitrile hydratase activator protein [53,54]. Nitrile hydratases function in the degradation of xenobiotic compounds, but they are also involved in tryptophan metabolism, specifically in the conversion of 3-indoleacetonitrile to indole-3-acetamide, which is a precursor of the plant hormone auxin [65,66]. SMa0044 has an unusual expression pattern in that it is expressed at a very low level in approximately half of the nodules tested (Table 3; Figure 4), but is expressed quite strongly by free-living *S. meliloti* on LBMC medium ( Additional file 5). SMa0044 is predicted to encode a member of the DUF2277 superfamily, which is has no known function [52].

## Conclusions

The goal of this study was to identify *S. meliloti* 1021 ORFs involved in host plant nodulation and nitrogen fixation. The comparative genomics method we employed was able to rediscover 19 ORFs that have previously been shown to be important for nodulation and/or nitrogen fixation. The earlier studies that identified these genes, in most cases, employed the classical bacterial genetic techniques of transposon mutagenesis, followed by strain isolation and phenotypic screening [11,67][68]. Our study identified 9 additional *S. meliloti* ORFs (out of the 13 we analyzed) that we have shown are expressed primarily in host plant nodules. However none of these newly identified ORFs were required for development of a functional symbiosis under the conditions we tested. Our results suggest that the accumulated transposon screens for essential *S. meliloti* nodulation/nitrogen fixation genes may be nearing saturation. However, the comparative genomics method described above might be very effective for identifying factors involved in the production of a phenotype common to a group of bacterial species that have not yet been studied by classical transposon mutagenesis screens.

## Competing interests

The authors declare that they have no competing interests.

## Authors’ contributions

KMJ conceived of the study, performed the genome comparisons, designed experiments, constructed bacterial mutant strains, performed experiments, interpreted results and drafted the manuscript. CQ designed experiments, constructed bacterial mutant strains, performed experiments, interpreted results and helped draft the manuscript. BKW constructed bacterial mutant strains, performed experiments, and helped draft the manuscript. OMD, JS, TEB, and MRL constructed bacterial mutant strains and performed experiments. All authors read and approved the final manuscript.

## Supplementary Material

Additional file 1 **Table S1.** Joint Genome Institute, Integrated Microbial Genomes Phylogenetic Profile search data on single genes. Click here for file

Additional file 2 **Table S2.** Primers used to amplify *S. meliloti* 1021 fragments for construction of insertion mutants and deletion mutants. Click here for file

Additional file 3 **Table S3.** Gene list of 139 ORFs compiled from search data in Additional file 1: Table S1. Click here for file

Additional file 4 **Free-living expression of β-glucuronidase (GUS) under the control of the promoters of the following ORFs:** A) clockwise from lower left—SMc01266; *greA* (positive control for GUS expression); *S. meliloti* 1021 wild type (negative control for GUS expression); SMb20431; SMa1334. (The cropped plate wedges in panel A are all from the same plate.) B) clockwise from lower right—SMc01986; SMc01562; SMc03964; *greA*; *S. meliloti* 1021; a second streak of SMc03964. C) (clockwise from left) *greA*; *S. meliloti* 1021; SMb20360 (two separate strains). Specific strain names are shown in the photo labels. The growth medium is LBMC, with streptomycin 500 ug/mL. Click here for file

Additional file 5 **Free-living expression of β-glucuronidase (GUS) under the control of the promoters of the following ORFs:** A) SMa0044. Multiple isolates of the SMa0044::GUS fusions are shown in comparison with *greA* (positive control for GUS expression) and *S. meliloti* 1021 wild type (negative control for GUS expression). B) SMc00135. Multiple isolates of the SMc00135::GUS fusions are shown in comparison with *greA* and *S. meliloti* 1021 wild type. C) the SMc01424-01422 operon. Multiple isolates of the SMc01424-01422: GUS fusions are shown in comparison with *greA* and *S. meliloti* 1021 wild type. The growth medium is LBMC, with streptomycin 500 ug/mL. GUS expression strains that were tested for nodule expression are denoted with an asterisk and are described in Tables 3 and 4. Click here for file
