# Supplementary figures and images for: A comparative genomics screen identifies a Sinorhizobium meliloti 1021 sodM-like gene strongly expressed within host plant nodules
Source: BMC Microbiol. 2012 May 15;12:74. doi: 10.1186/1471-2180-12-74 (PMC3462710; doi:10.1186/1471-2180-12-74)

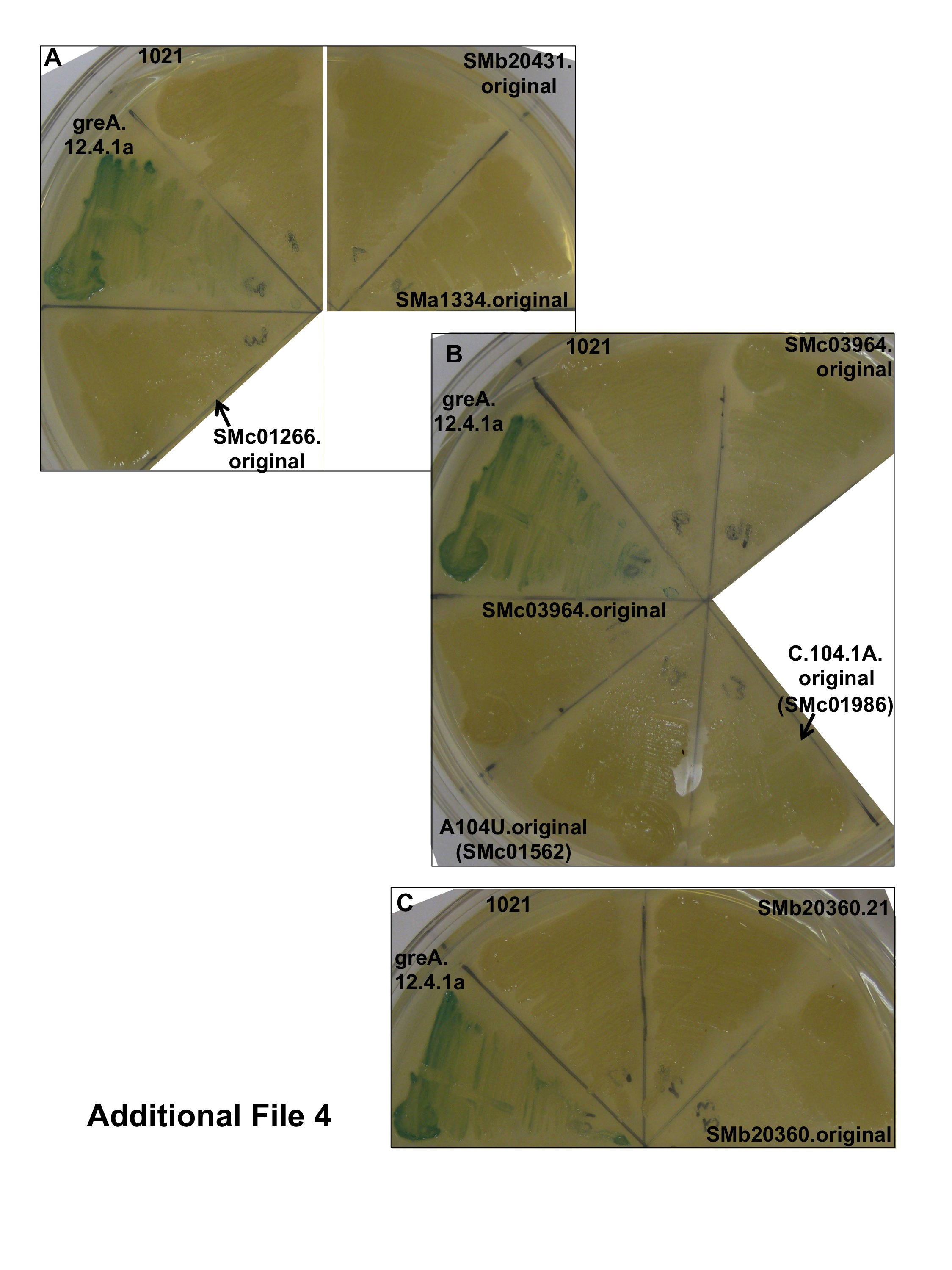

Supplement: Additional file 4 — Free-living expression of β-glucuronidase (GUS) under the control of the promoters of the following ORFs: A) clockwise from lower left—SMc01266; greA (positive control for GUS expression); S. meliloti 1021 wild type (negative control for GUS expression); SMb20431; SMa1334. (The cropped plate wedges in panel A are all from the same plate.) B) clockwise from lower right—SMc01986; SMc01562; SMc03964; greA; S. meliloti 1021; a second streak of SMc03964. C) (clockwise from left) greA; S. meliloti 1021; SMb20360 (two separate strains). Specific strain names are shown in the photo labels. The growth medium is LBMC, with streptomycin 500 ug/mL. [file 1471-2180-12-74-S4.jpeg]

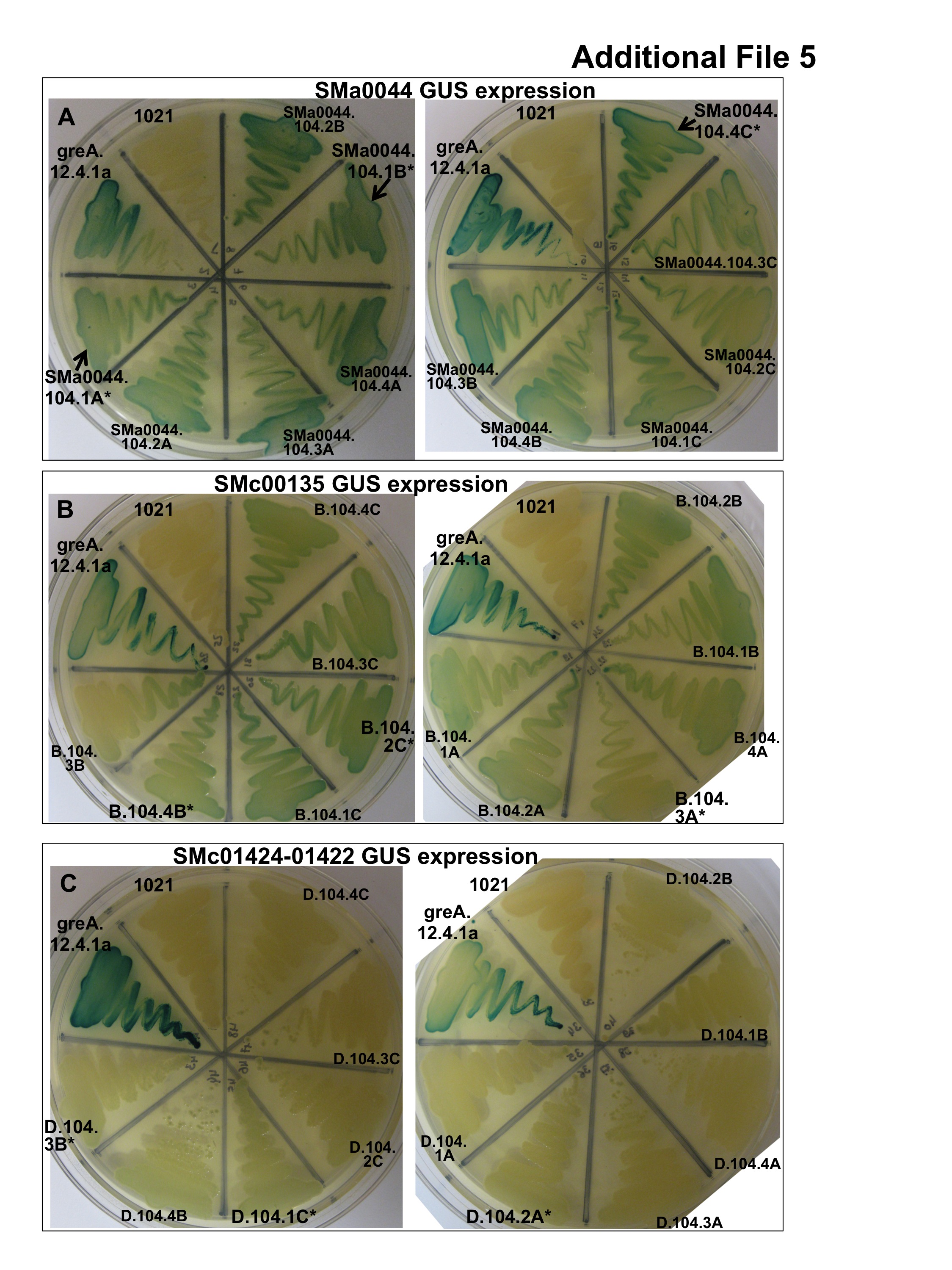

Supplement: Additional file 5 — Free-living expression of β-glucuronidase (GUS) under the control of the promoters of the following ORFs: A) SMa0044. Multiple isolates of the SMa0044::GUS fusions are shown in comparison with greA (positive control for GUS expression) and S. meliloti 1021 wild type (negative control for GUS expression). B) SMc00135. Multiple isolates of the SMc00135::GUS fusions are shown in comparison with greA and S. meliloti 1021 wild type. C) the SMc01424-01422 operon. Multiple isolates of the SMc01424-01422: GUS fusions are shown in comparison with greA and S. meliloti 1021 wild type. The growth medium is LBMC, with streptomycin 500 ug/mL. GUS expression strains that were tested for nodule expression are denoted with an asterisk and are described in Tables 3 and 4. [file 1471-2180-12-74-S5.jpeg]
